# Rice stripe virus activates the bZIP17/28 branch of the unfolded protein response signalling pathway to promote viral infection

**DOI:** 10.1111/mpp.13171

**Published:** 2021-12-11

**Authors:** Chenyang Li, Tianze Zhang, Yu Liu, Zongdi Li, Yaqin Wang, Shuai Fu, Yi Xu, Tong Zhou, Jianxiang Wu, Xueping Zhou

**Affiliations:** ^1^ State Key Laboratory of Rice Biology Institute of Biotechnology Zhejiang University Hangzhou China; ^2^ Key Laboratory of Food Quality and Safety Institute of Plant Protection Jiangsu Academy of Agricultural Sciences Nanjing China; ^3^ State Key Laboratory for Biology of Plant Diseases and Insect Pests Institute of Plant Protection Chinese Academy of Agricultural Sciences Beijing China; ^4^ Present address: Department of Plant Pathology College of Plant Protection Nanjing Agricultural University Nanjing 210095 China

**Keywords:** bZIP17/28, bZIP60, *Rice stripe virus*, unfolded protein response, viral infection

## Abstract

The unfolded protein response (UPR) plays important roles in plant virus infection. Our previous study has proved that rice stripe virus (RSV) infection elicits host UPR. However, the mechanism on how the UPR is triggered upon RSV infection remains obscure. Here, we show that the bZIP17/28 branch of the UPR signalling pathway is activated upon RSV infection in *Nicotiana benthamiana*. We found that membrane‐associated proteins NSvc2 and NSvc4 encoded by RSV are responsible for the activation of the bZIP17/28 branch. Ectopic expression of NSvc2 or NSvc4 in plant leaves induced the proteolytic processing of NbbZIP17/28 and up‐regulated the expression of UPR‐related genes. Silencing *NbbZIP17/28* significantly inhibited RSV infection. We show that RSV can specifically elicit the UPR through the bZIP17/28 branch, thus promoting virus infection of *N. benthamiana* plants.

## Graphical abstract

Rice stripe virus‐encoded membrane‐associated proteins NSvc2 and NSvc4 activate the bZIP17/28 branch of the unfolded protein response signalling pathway to promote viral infection.

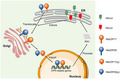

## 1 INTRODUCTION

In eukaryotes, the endoplasmic reticulum (ER) is the main protein factory for protein biosynthesis, folding, modification, quality control, and dispatch (Bao & Howell, 2017; Howell, 2013). Under stress conditions such as drought, salt, heat or pathogen invasion, the ER homeostasis is perturbed and unfolded proteins aggregate in the ER, which leads to ER stress and impairs the normal functions of this organelle (Howell, 2013; Walter & Ron, 2011). To relieve ER stress, the unfolded protein response (UPR) is induced. The UPR is a series of physiological processes aiming to restore ER homeostasis, including the halting of protein translation, the biogenesis of ER, the degradation of misfolded proteins, and the increase of molecular chaperones production (Howell, 2013; Walter & Ron, 2011).

Stress of ER is perceived and the signal is transmitted to the nucleus via the UPR signalling pathway. So far, two branches of this pathway have been well established in *Arabidopsis thaliana* (Bao & Howell, 2017). One of the branches involves the inositol‐requiring enzyme (IRE1), an ER transmembrane protein, and the basic leucine zipper 60 (bZIP60) transcription factor. When IRE1 senses ER stress, the *bZIP60* mRNA is spliced by IRE1, resulting in the excision of a 23 bp intron (Deng et al., 2011; Nagashima et al., 2011). The unspliced form of *bZIP60* mRNA [*bZIP60*(u)] encodes a transcription factor anchored at the ER membrane through its transmembrane domain (TMD), while splicing causes a frameshift eliminating the TMD, yielding a spliced form of *bZIP60* [*bZIP60*(s)] that encodes a nucleus‐targeted transcription factor (Deng et al., 2011; Nagashima et al., 2011). The other branch is involved in the bZIP17/bZIP28 transcription factors. The bZIP17/28 proteins are also anchored on the ER membrane through the TMDs and are retained by the interaction with the binding protein (BiP). Under the stress conditions, BiP is competed away by unfolded proteins in the ER lumen and disassociates from bZIP28 (Bao & Howell, 2017; Srivastava et al., 2013). Then, bZIP17/28 are transported from the ER to the Golgi apparatus, and cleaved by two Golgi resident proteases, Site‐1 and Site‐2 protease (S1P and S2P). The TMDs of bZIP17/28 are removed by proteolytic processing by S2P, resulting in the nucleus‐localized processed form of bZIP17/28 [bZIP17/28(p)] (Bao & Howell, 2017; Liu et al., 2007; Srivastava et al., 2013). The bZIP60(s) and bZIP17/28(p) are located in the nucleus, where they up‐regulate the expression of stress‐related genes to activate the UPR (Bao & Howell, 2017).

Viruses are obligate parasites that rely on their host living cells to complete DNA/RNA and protein synthesis (Ghazal et al., 2000; Xu et al., 2021; Zhou, 2021). Several studies have demonstrated that virus infections can induce ER stress and the UPR in plant cells, and the UPR plays important roles in virus infections (Gayral et al., 2020; Li et al., 2018, 2020, 2021; Luan et al., 2016; Zhang et al., 2015). Turnip mosaic virus (TuMV), potato virus X (PVX), and Plantago asiatica mosaic virus (PlAMV) infections induce the splicing of *bZIP60* mRNA through their membrane‐associated proteins (6K2 and TGBp3) and trigger the UPR (Gayral et al., 2020; Ye et al., 2011; Zhang et al., 2015). Silencing or knocking out the IRE1‐*bZIP60* branch inhibits viral infection (Gayral et al., 2020; Ye et al., 2011; Zhang et al., 2015). Up to now, some studies have confirmed the splicing of *bZIP60* mRNA upon virus infection, but it is still unclear whether bZIP17/28 has undergone proteolysis.

Rice stripe virus (RSV) is one of the most devastating rice viruses in East Asia (Wang et al., 2008; Xu et al., 2021). Rice plants infected with RSV typically exhibit symptoms affecting rice production seriously, such as chlorosis, discontinuous yellow stripes, necrosis in newly emerged leaves, and growth retardation (Wang et al., 2008; Xu et al., 2021). *Rice stripe virus* is a segmented single‐stranded negative‐sense RNA plant virus species, in the *Tenuivirus* genus of the family *Phenuiviridae*. The viral genome consists of four RNA molecules, which range in size from approximately 8.9 to 2.1 kb (Adams et al., 2017; Xiong et al., 2008; Xu et al., 2021). The viral genome encodes seven proteins, among which NSvc2 and NSvc4 (encoded by the complementary strands of RNA2 and RNA4, respectively) are membrane‐associated proteins (Xiong et al., 2008; Xu & Zhou, 2012; Yao et al., 2014). *Nicotiana benthamiana* is a widely used experimental host in plant virology (Goodin et al., 2008). Previous work has shown that RSV can infect *N. benthamiana* by mechanical inoculation, causing symptoms including dwarfing, vein yellowing, and leaf curling (Kong et al., 2014; Xu & Zhou, 2012). *N. benthamiana* has become one of the model plants for studying the pathogenicity and host resistance mechanisms of RSV (Chen et al., 2020; Fu et al., 2018; Jiang et al., 2021; Kong et al., 2014; Li et al., 2021). Our previous study has proved that RSV infection elicits the UPR and the UPR is indispensable for viral infection (Li et al., 2021). However, it is still unclear how RSV triggers UPR, and whether NSvc2 or NSvc4 participates in the induction of UPR.

In the present study, we demonstrate that RSV infection activates the bZIP17/28 branch rather than the IRE1‐*bZIP60* branch. Expressing NSvc2 or NSvc4 in *N. benthamiana* leaves induces the proteolytic processing of bZIP17/28 and elicits the UPR. Silencing the bZIP17/28 branch can delay RSV infection and viral symptom development. Our study reveals that RSV selectively induces UPR through the bZIP17/28 branch, which promotes virus infection, and may provide valuable clues for the further study on the role of the UPR in plant virus infections.

## 2 RESULTS

### 2.1 *NbbZIP60* mRNA is spliced during ER stress, and RSV infection has no effect on the splicing of *NbbZIP60* mRNA in *N. benthamiana*

**FIGURE 1:**
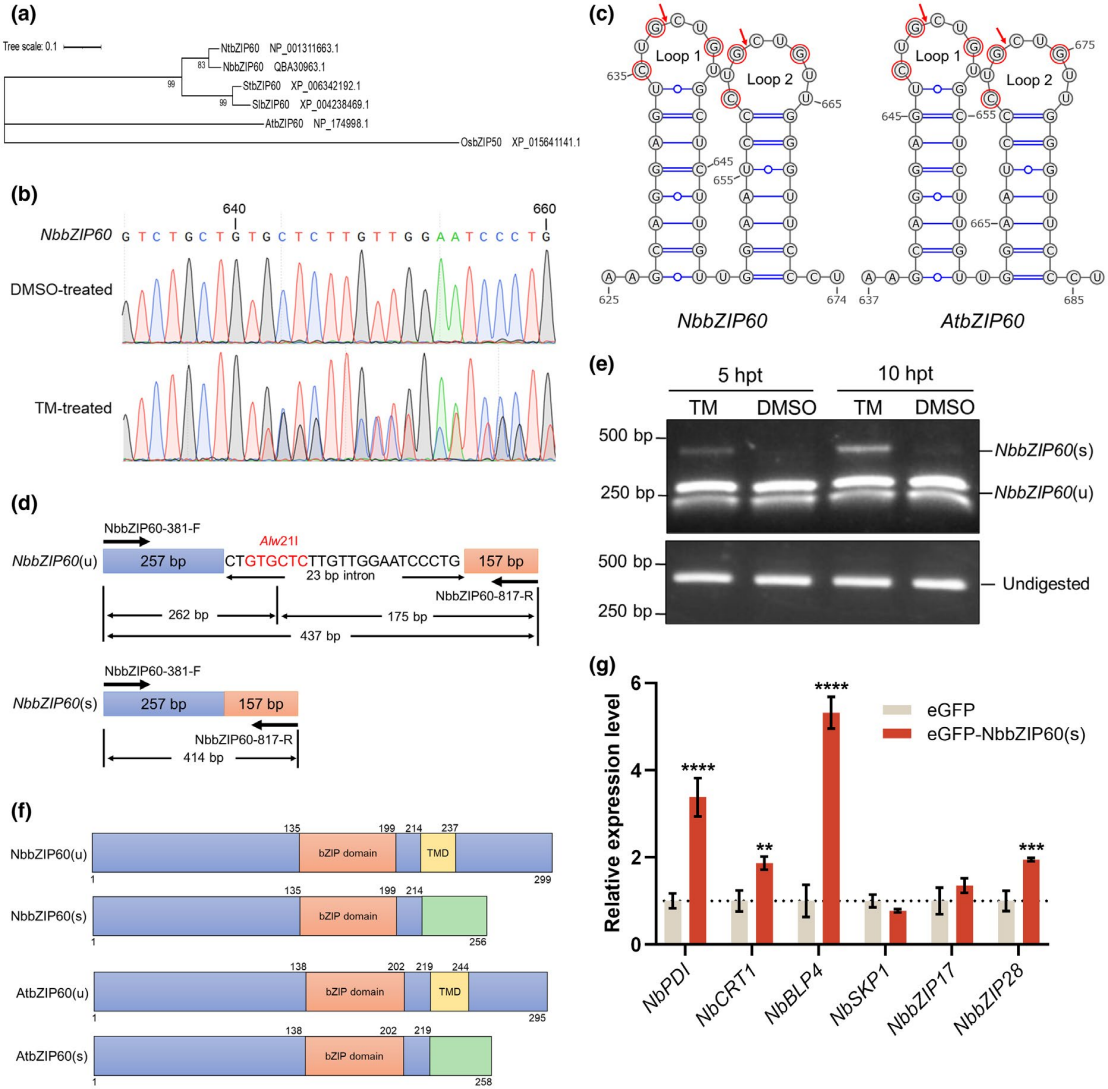
The mRNA of *NbbZIP60* is spliced upon endoplasmic reticulum (ER) stress. (a) Phylogenetic analysis of bZIP60 proteins in *Nicotiana benthamiana*, tobacco (*Nicotiana tabacum*), tomato (*Solanum lycopersicum*), potato (*Solanum tuberosum*), *Arabidopsis thaliana*, and rice (*Oryza sativa*). The phylogenetic tree was constructed using the maximum‐likelihood method and JTT matrix‐based model. The tree is drawn to scale and the bootstrap test (1000 replicates) was performed. The GenBank accession number of each sequence is shown next to the protein name. (b) Sanger sequencing results of the *NbbZIP60* cDNA in leaves of *N. benthamiana* treated with dimethyl sulfoxide (DMSO) or tunicamycin (TM). The *N. benthamiana* leaves were treated with 5 μg/ml TM diluted in double‐deionized water or an equal volume of DMSO as control. The total RNA of the leaf samples was extracted at 10 h posttreatment (hpt) for reverse transcription (RT)‐PCR, and the PCR products were subjected to Sanger sequencing. (c) Twin hairpin loop structures in *NbbZIP60* and *AtbZIP60* mRNAs at the splicing sites. Twin hairpin loop structures were predicted at the *NbbZIP60* and *AtbZIP60* mRNA splicing sites using the mfold web server, and the Δ*G* of these two structures are both −21.66 kcal/mol. Each of the two loops contains three conserved bases (red); red arrows indicate the predicted cleavage sites. (d) The schematic representation of the *bZIP60* splicing assay. The primers set flanking the 23‐bp intron to amplify the cDNA of *NbbZIP60*(u) (top) and *NbbZIP60*(s) (bottom). The *Alw*21I cleavage site in the 23‐bp intron is indicated in red; the length of each product is labelled. (e) The splicing assays of *NbbZIP60* in the TM‐ or DMSO‐treated *N. benthamiana* leaves. The leaves were treated with 5 μg/ml TM or an equal volume of DMSO as control. The total RNA of leaf samples was extracted at 5 and 10 hpt for the *bZIP60* splicing assays. The bands of *NbbZIP60*(s), *NbbZIP60*(u), and undigested products are labelled on the right; the DNA sizes are indicated on the left. (f) Conserved domain predictions of NbbZIP60(u), NbbZIP60(s), AtbZIP60(u), and AtbZIP60(s). The predicted domains and their positions are labelled: orange box, bZIP domain; yellow box, transmembrane domain (TMD); green box, new sequence caused by splicing. (g) The RT‐quantitative PCR (RT‐qPCR) analyses of the unfolded protein response (UPR)‐related genes when expressing eGFP‐NbbZIP60(s). The eGFP‐NbbZIP60(s) or eGFP (control) was expressed in *N. benthamiana* leaves by agroinfiltration. The total RNA of the leaf samples was extracted for RT‐qPCR analyses at 48 h postinfiltration. *NbActin* served as an internal reference in relative quantification. The values represent the means of the expression levels ± *SD* relative to the eGFP‐expressing leaves (*n* = 3 biological replicates). The values were analysed by analysis of variance followed by Dunnett's test, and asterisks denote significant differences between eGFP‐NbbZIP60s‐ and eGFP‐expressing leaves (two‐sided, **p < 0.01, ***p < 0.001, ****p < 0.0001)

Previous studies have found that in *A. thaliana*, *bZIP60* mRNA is spliced by IRE1 upon ER stress and viral infections of TuMV, PVX, and PlAMV, which leads to the UPR (Deng et al., 2011; Gaguancela et al., 2016; Nagashima et al., 2011; Zhang et al., 2015). *bZIP60* has been used as a indicative marker gene of the UPR (Li et al., 2018, 2020; Sun et al., 2013; Ye et al., 2011). Moreover, the orthologs of *AtbZIP60* in other plants, such as *SlbZIP60* in tomato (*Solanum lycopersicum*) and *OsbZIP50* in rice (*Oryza sativa*), have been identified and proved to be spliced upon ER stress (Hayashi et al., 2012; Kaur & Kaitheri Kandoth, 2021; Lu et al., 2012). To confirm whether *NbbZIP60* is the ortholog of these well‐characterized genes, the protein sequences of bZIP60 in *A. thaliana*, *Nicotiana tabacum*, tomato, potato (*Solanum tuberosum*), and rice were retrieved from the GenBank database for phylogenetic analysis. The results showed that NbbZIP60 clustered with bZIP60s from other plants in the Solanaceae family and was more distantly related to AtbZIP60 and OsbZIP50 (Figure 1a), suggesting *NbbZIP60* is the ortholog of *bZIP60* genes.

Then, the *NbbZIP60* sequence (GenBank accession number MG753987.1) was cloned to perform the following experiments. Tunicamycin (TM) is a chemical used to trigger ER stress and activate the UPR (Bukau et al., 2006; Liu et al., 2007; Nagashima et al., 2011). To find out whether the mRNA of *NbbZIP60* can be spliced during ER stress, the leaves of *N. benthamiana* were treated with TM or dimethyl sulfoxide (DMSO) (control). Ten hours later, the cDNA of *NbbZIP60* was amplified and sequenced. The sequencing result of TM‐treated leaves showed that there were multiple peaks at 642 bp of the coding sequence (CDS), while the sequencing peaks of DMSO‐treated leaves were still sharp (Figure 1b), suggesting that the mRNA of *NbbZIP60* may be spliced upon TM treatment. Then, the two forms of *NbbZIP60* were cloned and designated as *NbbZIP60* (unspliced, *NbbZIP60*u) and *NbbZIP60* (spliced, *NbbZIP60*s), which has a deletion of 23 bp (638–660 bp of the CDS), resulting in a frameshift mutation of the CDS. The secondary structure of the splicing sites of *NbbZIP60* mRNA was predicted to fold as a conserved twin hairpin loop, and the conserved splice sites (CUG/CUG) were also found in each loop (Figure 1c) (Nagashima et al., 2011; Zhang et al., 2015). Next, the *NbbZIP60*(s) was monitored following an approach based on restriction enzyme digestion described previously (Lipatova & Segev, 2015; Moreno et al., 2012). The PCR products of *NbbZIP60* CDS (position 381–817 bp) were digested by *Alw*21I, which could only cleave the 23‐bp intron of *NbbZIP60*(u) (Figure 1d). We found that all the amplification products were digested into 262‐bp and 175‐bp fragments in DMSO‐treated leaves, while TM treatment resulted in the appearance of a 414‐bp band (Figure 1e), indicating that the *NbbZIP60*(u) was processed into *NbbZIP60*(s) after the TM treatment. Next, the conserved bZIP domain was predicted in both NbbZIP60(u) and NbbZIP60(s), and a TMD was predicted at the C‐terminus of NbbZIP60(u). In NbbZIP60(s) the TMD is replaced by a new amino acid sequence caused by the frameshifting mutation, which is similar to AtbZIP60(s) (Figure 1f). The following reverse transcription quantitative PCR (RT‐qPCR) analysis showed that compared with the leaves expressing eGFP, the transcription levels of UPR‐related genes in the leaves expressing eGFP‐NbbZIP60(s) was significantly up‐regulated (Figure 1g), suggesting that NbbZIP60(s) is a regulator of the UPR in *N. benthamiana*.

**FIGURE 2:**
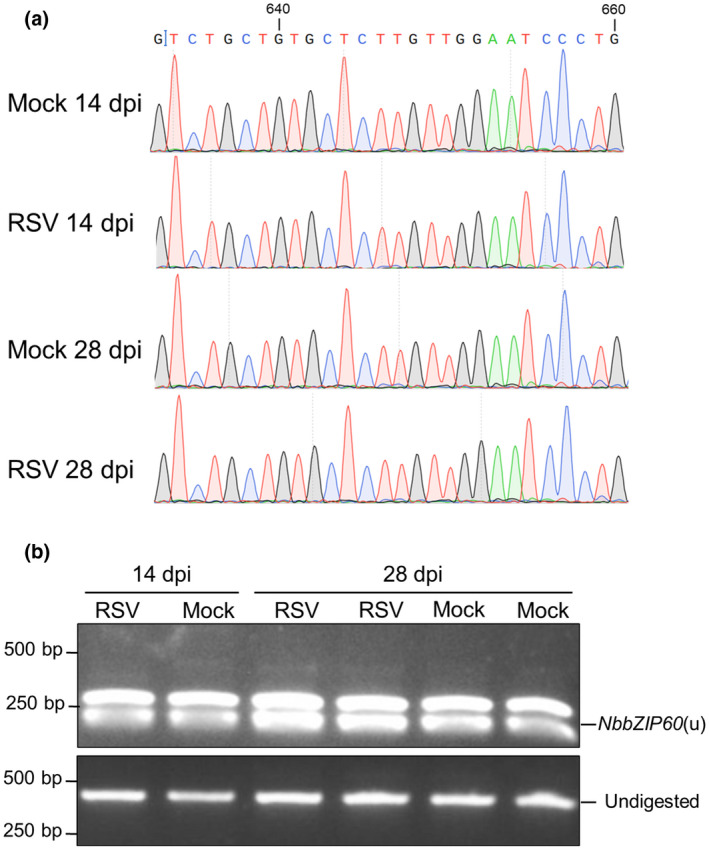
RSV infection has no effect on the splicing of *NbbZIP60* mRNA. (a) Sanger sequencing results of *NbbZIP60* cDNA in RSV‐infected or mock‐infected *Nicotiana benthamiana* plants. The total RNA of RSV‐infected or mock control plants was extracted at 14 days postinoculation (dpi) or 28 dpi for reverse transcription (RT)‐PCR and sequencing. (b) The splicing assays of *NbbZIP60* in RSV‐infected *N. benthamiana* plants. The RT‐PCR products in (a) were subjected to flanking assays. The bands of *NbbZIP60*(u) and undigested products are labelled on the right; the DNA sizes are indicated on the left

Our previous study demonstrated that RSV infection elicits the UPR of host cells (Li et al., 2021). To confirm whether the *NbbZIP60* mRNA is spliced upon RSV infection, we amplified the fragment of *NbbZIP60* cDNA through RT‐PCR from RSV‐infected *N. benthamiana* plants or mock controls, and then the amplification products were purified for sequencing. Surprisingly, we did not find any obvious multiple peaks in the sequencing results (Figure 2a), which suggests the mRNA of *NbbZIP60* might not be spliced upon RSV infection. The purified amplification products were also digested by *Alw*21I and then separated by gel electrophoresis. As shown in Figure 2b, all the PCR products were completely digested, indicating that the mRNA of *NbbZIP60* was not spliced. These findings indicate that RSV might elicit the UPR through other branches rather than the IRE1‐*bZIP60* branch.

### 2.2 NbbZIP17/28 are proteolytically processed upon ER stress in *N. benthamiana*

**FIGURE 3:**
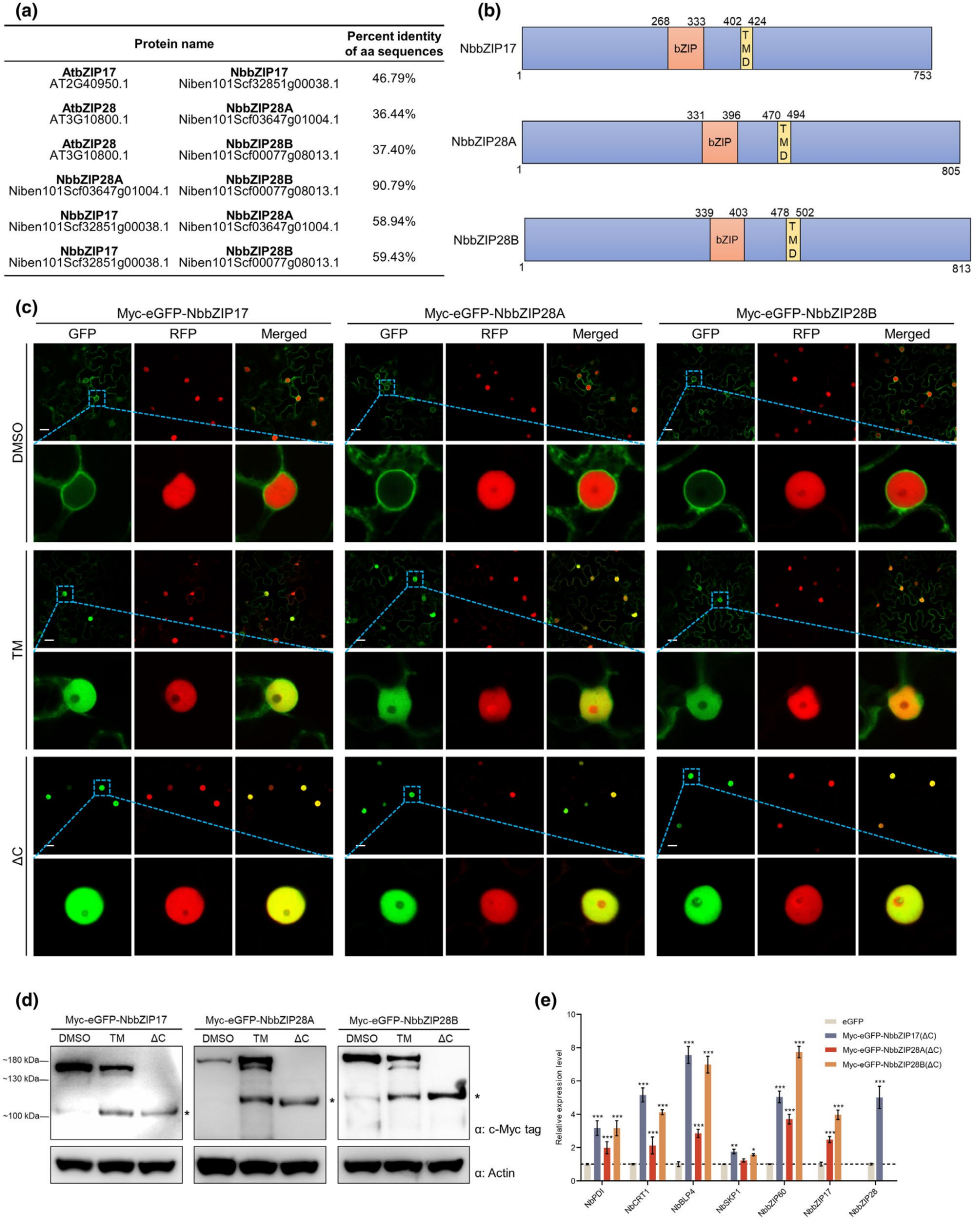
NbbZIP17/28 are proteolytically processed upon endoplasmic reticulum (ER) stress in *Nicotiana benthamiana*. (a) The amino acid sequence comparisons of AtbZIP17/28 between NbbZIP17/28. (b) Conserved domain predictions of NbbZIP17/28A/28B. The predicted domains and their positions are labelled on the proteins: orange box, bZIP domain; yellow box, transmembrane domain (TMD). (c) Confocal images of *N. benthamiana* leaves expressing Myc‐eGFP‐NbbZIP17/28A/28B by agroinfiltration (OD_600_ = 0.04, 0.2, 0.08, respectively, and treated with dimethyl sulfoxide [DMSO] or tunicamycin [TM]) and their C‐terminal deleted mutants (ΔC). The images were taken at 48 h after agroinfiltration. The TM was diluted to 1.5 μg/ml and infiltrated into leaves 4 h before observation. RFP‐H2B was expressed as a nucleus marker. The magnified areas are indicated by blue boxes. Bars, 20 μm. (d) Western blot analyses of *N. benthamiana* leaves expressing Myc‐eGFP‐NbbZIP17/28A/28B (treated with DMSO or TM) and their C‐terminal deleted mutants (ΔC). The leaf samples in (c) were harvested and total protein was extracted for western blot analyses. NbActin was used as a loading control. Molecular mass markers are indicated on the left. (e) The reverse transcription‐quantitative PCR (RT‐qPCR) analyses of the unfolded protein response (UPR)‐related genes when expressing Myc‐eGFP‐NbbZIP17/28A/28B(ΔC). The Myc‐eGFP‐NbbZIP17(ΔC), Myc‐eGFP‐NbbZIP28A(ΔC), Myc‐eGFP‐NbbZIP28B(ΔC) or eGFP (control) was expressed in *N. benthamiana* leaves. The total RNA of the leaf samples was extracted for RT‐qPCR analyses at 48 h postinoculation. *NbActin* served as an internal reference in relative quantification. The values represent the means of the expression levels ± *SD* relative to the eGFP‐expressing leaves (*n* = 3 biological replicates). The values were analysed by analysis of variance followed by Dunnett's test, and asterisks denote significant differences between Myc‐eGFP‐NbbZIP17/28(ΔC) and eGFP‐expressing leaves (two‐sided, *p < 0.05, **p < 0.01, ***p < 0.001)

The above results indicate that RSV infection may not elicit the UPR through the IRE1‐*bZIP60* branch. We assume that RSV infection might activate the bZIP17/28 branch. However, *bZIP17*/*28* have not been cloned and identified in *N. benthamiana*. To find the homologs of *AtbZIP17* and *AtbZIP28* in *N. benthamiana*, a BLASTp search was performed using the amino acid sequences of AtbZIP17 or AtbZIP28 as the query in the *N. benthamiana* genome database. Only three related proteins were found, and the results showed that AtbZIP17 was similar to Niben101Scf32851g00038.1, while AtbZIP28 was similar to Niben101Scf03647g01004.1 and Niben101Scf00077g08013.1 (Figure S1a,b and Figure 3a). Moreover, we also found that the protein sequences of Niben101Scf03647g01004.1 and Niben101Scf00077g08013.1 were highly similar, and shared more than 90% sequence identity, while they are more distantly related to Niben101Scf32851g00038.1 (<60% sequence identity) (Figure 3a). We postulate that Niben101Scf03647g01004.1 and Niben101Scf00077g08013.1 are from the two ancestries of *N. benthamiana*. Therefore, we tentatively designated Niben101Scf32851g00038.1 as *NbbZIP17*, Niben101Scf03647g01004.1 and Niben101Scf00077g08013.1 as *NbbZIP28A* and *NbbZIP28B*, respectively.

The analysis of conserved domains through InterProScan revealed that NbbZIP17/28A/28B encoded the bZIP domains and TMDs (Figure 3b), suggesting that NbbZIP17/28A/28B may also be anchored at the ER. To test this, N‐terminal tagged Myc‐eGFP‐NbbZIP17/28A/28B fusion proteins were constructed and expressed in *N. benthamiana* leaves by agroinfiltration. To our surprise, confocal observation showed that Myc‐eGFP‐NbbZIP17/28A/28B localized in both the cytoplasm and the nucleus (Figure S2). We speculated that this auto‐activation was due to the overexpression of Myc‐eGFP‐NbbZIP17/28A/28B, and the nuclear localization disappeared when NbbZIP17/28A/28B were expressed at a moderate level by diluting the *Agrobacterium* suspensions to OD_600_ = 0.04, 0.2, 0.08, respectively (Figure 3c). Then, the leaves expressing Myc‐eGFP‐NbbZIP17/28A/28B were treated with TM, and eGFP fluorescence signals were detected in the nucleus at 4 h posttreatment (hpt) (Figure 3c), indicating that NbbZIP17/28A/28B could respond to ER stress and be transported to the nucleus. Moreover, we also found that the C‐terminal truncated mutants NbbZIP17/28A/28B(ΔC), which lacked TMDs, localized in the nucleus exclusively (Figure 3c). These results suggest NbbZIP17/28A/28B may also undergo a proteolytic process that cleaves TMDs and C‐terminal parts from the proteins during ER stress.

To confirm the proteolytic processing of NbbZIP17/28A/28B, the total protein was extracted and used for western blot analyses. The results of NbbZIP17/28A/28B proteins are similar: a cleaved band appeared after the TM treatment, and the molecular mass of this band was approximately similar to the mass of the C‐terminal truncated mutant (Figure 3d), which indicated that NbbZIP17/28A/28B were proteolytically cleaved at their C‐termini to remove the TMDs upon ER stress. We also noticed that the actual molecular mass of these proteins was larger than the estimated mass, which may be due to the glycosylation of these proteins (Liu et al., 2007). Moreover, RT‐qPCR analyses showed that the UPR‐related genes were significantly up‐regulated in Myc‐eGFP‐NbbZIP17/28A/28B(ΔC) leaves compared with those expressing eGFP (Figure 3e), suggesting that the processed NbbZIP17/28A/28B are capable of activating the expression of UPR‐related genes.

### 2.3 RSV infection activates the bZIP17/28 branch of the UPR in *N. benthamiana*

**FIGURE 4:**
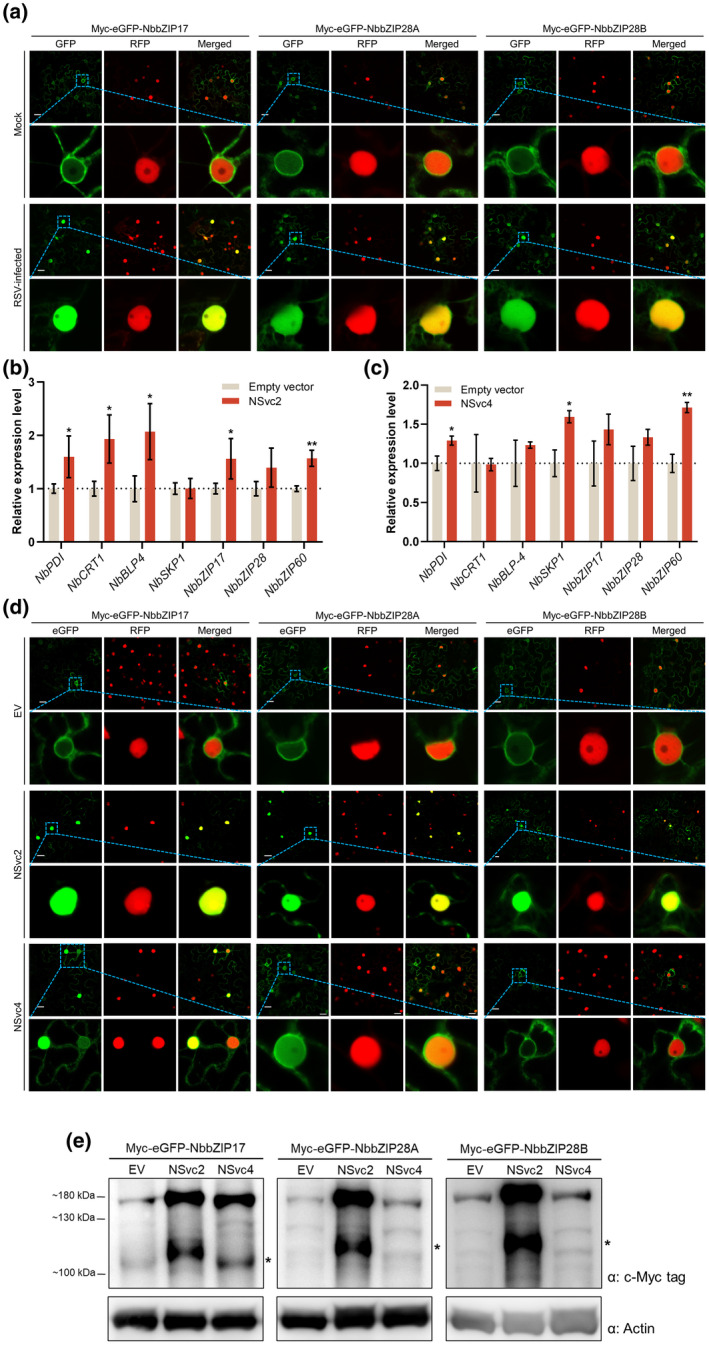
RSV infection activates the bZIP17/28 branch of the unfolded protein response (UPR). (a) Confocal images of RSV‐infected *Nicotiana benthamiana* leaves expressing Myc‐eGFP‐NbbZIP17/28A/28B. Myc‐eGFP‐NbbZIP17/28A/28B were expressed in RSV‐infected *N. benthamiana* leaves at 12 days postinoculation (dpi), and images were taken 48 h later. RFP‐H2B was expressed as nucleus marker. The magnified areas are indicated by blue boxes. Bars, 20 μm. (b, c) The reverse transcription quantitative PCR (RT‐qPCR) analyses of the UPR‐related genes when expressing NSvc2 (b) or NSvc4 (c). NSvc2, NSvc4 or empty control was expressed in *N. benthamiana* leaves through agroinfiltration. The total RNA of the leaf samples was extracted for RT‐qPCR analyses at 60 h postinfiltration (hpi). *NbActin* served as an internal reference in relative quantification. The values represent the means of the expression levels ± *SD* relative to the empty vector expressed leaves (*n* = 3 biological replicates). The values were analysed by analysis of variance followed by Dunnett's test, and asterisks denote significant differences between NSvc2 or NSvc4 and empty vector‐expressing leaves (two‐sided, *p < 0.05, **p < 0.01). (d) Confocal images of *N. benthamiana* leaves co‐expressing Myc‐eGFP‐NbbZIP17/28A/28B with NSvc2 or NSvc4. Myc‐eGFP‐NbbZIP17/28A/28B were co‐expressed with NSvc2, NSvc4 or empty vector control through agroinfiltration. The images were taken at 48 hpi. (e) Western blot analyses of *N. benthamiana* leaves co‐expressing Myc‐eGFP‐NbbZIP17/28A/28B with NSvc2, NSvc4 or empty vector control. The leaf samples in (d) were harvested and total protein was extracted for western blot analyses. Actin was used as a loading control. Molecular mass markers are indicated on the left

To determine whether RSV infection could activate the bZIP17/28 branch, the Myc‐eGFP‐NbbZIP17/28A/28B proteins were expressed in the leaves of RSV‐infected or healthy *N. benthamiana* plants. At 48 h postinoculation (hpi), we observed the leaves with a confocal microscope and found that Myc‐eGFP‐NbbZIP17/28A/28B were located in the nucleus and the cytoplasm of RSV‐infected leaves, but only in the cytoplasm in healthy leaves (Figure 4a), suggesting that the bZIP17/28 branch is activated upon RSV infection.

Previous studies found that viral membrane‐associated proteins might be inducers of the UPR (Ye et al., 2011; Zhang et al., 2015). To test whether RSV‐encoded membrane‐associated proteins (NSvc2 and NSvc4) (Xu & Zhou, 2012; Yao et al., 2014) can elicit the UPR, the NSvc2 or NSvc4 was expressed in *N. benthamiana* leaves individually through agroinfiltration, and total RNA was extracted for RT‐qPCR analyses at 60 hpi. Figure 4b,c shows that expressing NSvc2 or NSvc4 could up‐regulate the expression of the UPR‐related genes compared with the empty vector control. Also, other RSV‐encoded proteins were tested, and none of them elicited the UPR (Figure S3), indicating that NSvc2 and NSvc4 are the RSV‐encoded inducers of the UPR. Next, we wondered if NSvc2 and NSvc4 could activate the bZIP17/28 branch. NSvc2 or NSvc4 was co‐expressed with Myc‐eGFP‐NbbZIP17/28A/28B in *N. benthamiana* leaves. As shown in Figure 4d, expressing NSvc2 significantly increased the fluorescence intensities of Myc‐eGFP‐NbbZIP17/28A/28B in the nucleus. Expressing NSvc4 could also induce the nuclear localization of Myc‐eGFP‐NbbZIP17, although not as strongly as that in NSvc2‐expressed leaves. However, we noticed that Myc‐eGFP‐NbbZIP28A/B did not translocate to the nucleus when co‐expressed with NSvc4, which suggested that NSvc4 might be a weaker inducer of the UPR compared with NSvc2. The western blot analyses also indicated that expressing NSvc2 induced the cleavage of NbbZIP17/28A/28B, while NSvc4 only facilitated the cleavage of NbbZIP17 (Figure 4e). Moreover, NbbZIP17/28 accumulated dramatically when co‐expressed with NSvc2, implying that NSvc2 might regulate the protein stability of NbbZIP17/28. However, NSvc4 only increased the accumulation of NbbZIP17. These findings indicate that RSV infection may elicit the UPR through the bZIP17/28 branch in *N. benthamiana*.

### 2.4 Silencing *NbbZIP17/28* inhibits the infection of RSV

**FIGURE 5:**
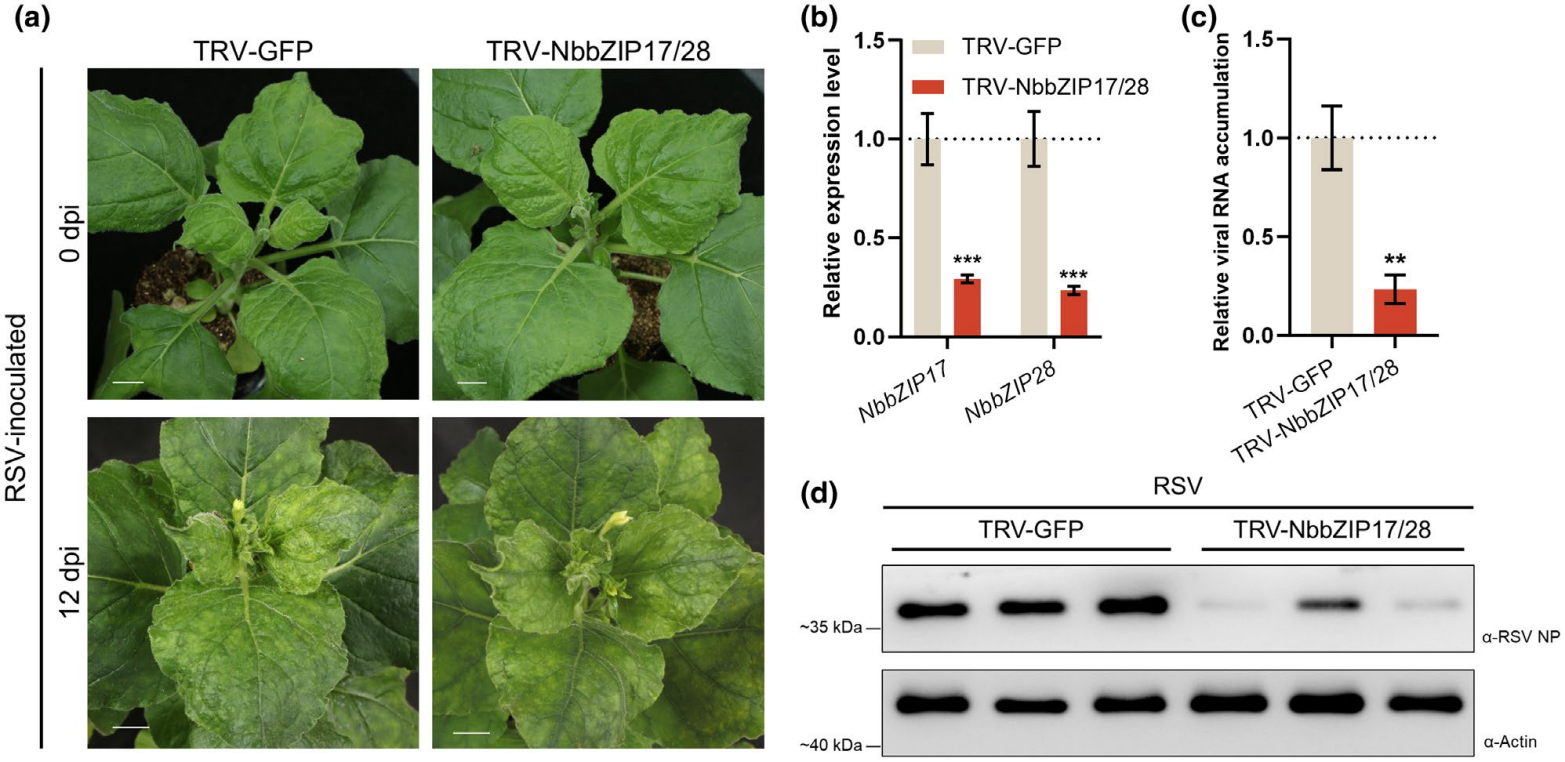
Silencing *NbbZIP17/28* inhibits the infection of RSV. (a) The viral symptom of RSV on *Nicotiana benthamiana* plants pre‐inoculated with TRV‐GFP (control) or TRV‐NbbZIP17/28. Bars, 1 cm. (b) Reverse transcription quantitative PCR (RT‐qPCR) analysis of the silencing efficiency of *NbbZIP17/28* in TRV‐NbbZIP17/28 pre‐inoculated *N. benthamiana*. The total RNA of the leaf samples from TRV‐GFP and TRV‐NbbZIP17/28 pre‐inoculated *N. benthamiana* plants was extracted for RT‐qPCR analyses at 10 days postinoculation (dpi). *NbActin* served as an internal reference in relative quantification. The values represent the means of the expression levels ± *SD* relative to the TRV‐GFP control plants (*n* = 3 biological replicates). Data were analysed by Student's *t* test, and asterisks denote significant differences between TRV‐NbbZIP17/28 and TRV‐GFP pre‐inoculated plants (two‐sided, ***p < 0.01). (c) RT‐qPCR analysis of RSV viral RNA accumulation. Total RNA of leaf samples in (a) at 12 dpi was extracted for RT‐qPCR analysis. *NbActin* served as an internal reference in relative quantification. The values represent the means of relative expression levels ± *SD* relative to the mock plants (*n* = 3 biological replicates). Data were analysed by Student's *t* test, and asterisks denote significant differences between RSV‐infected and mock plants (two‐sided, **p < 0.01). (d) Western blot analysis of RSV nucleocapsid protein (NP) accumulation. The leaf samples in (a) at 12 dpi of RSV were harvested and total protein was extracted for western blot analyses. NbActin was used as a loading control. Molecular mass markers are indicated on the left

In our previous study, we demonstrated that the UPR is essential for the infection of RSV, and that treatment with 4‐phenylbutyric acid (4‐PBA), a chemical chaperone that can alleviate ER stress and inhibit the UPR, alleviates viral infection of *N. benthamiana* (Li et al., 2021; Ranga Rao et al., 2018; Zhang et al., 2015). To confirm the role of bZIP17/28 in RSV infection, we silenced *NbbZIP17/28* by using virus‐induced gene silencing (VIGS) based on tobacco rattle virus (TRV) and then challenged the silenced plants with RSV. A fragment combining 835–1049 bp of the *NbbZIP17* open reading frame (ORF) and 1717–1918 bp of the *NbbZIP28A* ORF was inserted into the pTRV2 vector, and SGN VIGS Tool (https://vigs.solgenomics.net/) analysis showed that the siRNA derived from this fragment only targeted *NbbZIP17/28A/28B* (Figure S4). The *NbbZIP17/28*‐silenced plants did not show any obvious phenotype compared with the plants infected with the TRV‐GFP control at 10 days after TRV pre‐inoculation (Figure 5a, first row), and the silencing efficiency was detected through RT‐qPCR (Figure 5b). Then, these plants were challenged with RSV by mechanical inoculation, and noticeable yellowing of leaf veins was observed in the TRV‐GFP control plants, while this symptom was milder in the TRV‐NbbZIP17/28 plants at 12 days postinoculation (dpi) (Figure 5a, second row). The accumulation of the RSV RNA and nucleocapsid protein in the *NbbZIP17/28*‐silenced plants was significantly lower than that in the TRV‐GFP‐infected plants, correlating with viral symptoms (Figure 5c,d). These results demonstrate that silencing *NbbZIP17/28* can delay RSV infection and symptom development, and RSV may elicit the UPR through the *NbbZIP17/28* branch to promote its infection.

## 3 DISCUSSION

The ER is the main protein synthesis factory in the cell, and plant viruses rely on host protein synthesis machinery to produce the proteins that are essential for their life cycle. In addition, the replication and intracellular transport of some viruses involve the host ER endomembrane system (Verchot, 2016). The UPR is a series of cell biological processes that are essential for cells to overcome abiotic or biotic stresses (Howell, 2013; Walter & Ron, 2011). Many plant viruses are capable of eliciting the UPR in host cells, and the UPR has been found to play important roles in the infection of plant viruses (Li et al., 2018; Luan et al., 2016; Zhang et al., 2015). The PVX‐encoded TGBp3 protein is responsible for eliciting the UPR (Ye et al., 2011). TuMV‐encoded 6K2 protein triggers the UPR, which activates the NBR1‐mediated selective autophagy to help viral infection and accumulation (Li et al., 2020; Zhang et al., 2015). In our previous study, we proved that the RSV‐induced UPR also regulates RSV infection (Li et al., 2021). However, it is still unclear how RSV infection elicits host UPR.

Stress conditions in the ER are communicated to the nucleus via the UPR signalling pathway. Two branches of this signalling pathway have been identified in plants, namely, the IRE1‐*bZIP60* and bZIP17/28 branches (Bao & Howell, 2017). Upon ER stress, *bZIP60* mRNA is spliced by IRE1, resulting in nuclear localized bZIP60(s) protein, thus up‐regulating the expression of UPR‐related genes. bZIP17/28 proteins are proteolytically processed to bZIP17/28(p), which are translocated to the nucleus to activate the UPR. *bZIP60* is spliced upon potyvirus (TuMV) or potexvirus (PVX and PlAMV) infections (Gaguancela et al., 2016; Gayral et al., 2020; Zhang et al., 2015). Although some studies have found that the bZIP17/28 branch is also related to the infection by tobacco mosaic virus (TMV), cucumber mosaic virus (CMV), TuMV and PlAMV (Gayral et al., 2020; Li et al., 2018; Shen et al., 2017), whether bZIP17/28 are proteolytically processed responding to viral infection is largely unknown. In our study, the mRNA of *NbbZIP60* remained unspliced upon RSV infection (Figure 2a,b), which suggests that RSV infection may not depend on the IRE1‐*bZIP60* branch to elicit the UPR. Moreover, we also found the alternative bZIP17/28 branch of the UPR in *N. benthamiana* (Figure 3a,b) and provided evidence that NbbZIP17, NbbZIP28A and NbbZIP28B are the sensors and regulators of the UPR signalling pathway (Figure 3c–e). RSV infection led to the translocation of NbbZIP17/28A/28B to the nucleus (Figure 4a). Transiently expressing NSvc2 or NSvc4 in *N. benthamiana* leaves up‐regulated the expression of UPR‐related genes (Figure 4b,c) and promoted the production of NbbZIP17(p) or NbbZIP28(p) for cell nuclear localization (Figure 4d,e). These results indicate that RSV infection elicits the UPR through the bZIP17/28 branch in *N. benthamiana*, and RSV‐encoded membrane‐associated proteins NSvc2 and NSvc4 are the inducers of the UPR. However, NSvc4 only promoted the cleavage of NbbZIP17, while NSvc2 induced the processing of all NbbZIP17/28A/28B proteins (Figure 4e), suggesting that NSvc4 is a weaker inducer of the UPR than NSvc2.

It is also noteworthy that the expression of NSvc2 or NSvc4 significantly increased the protein accumulation levels of transiently expressed NbbZIP17 or NbbZIP28A/28B (Figure 4e), implying there might be a mechanism that regulates the protein stability of NbbZIP17/28A/28B in plants. However, we have not found any studies examining the turnover of bZIP17/28 in plants. Liu et al. found that the protein accumulation levels of AtbZIP17/28 are relatively lower than that of the cleaved AtbZIP17/28(p) products, and they suspected that the uncleaved precursor might turnover under nonstressed conditions (Liu et al., 2007). In our study, NSvc2 and NSvc4 inhibited the degradation of uncleaved NbbZIP17/28, resulting in the accumulation of the unprocessed precursors. Interestingly, we found that overaccumulation of NbbZIP17/28A/28B caused the auto‐activation of these proteins under normal conditions (Figure S2). Therefore, we speculate that NSvc2 and NSvc4 may activate the bZIP17/28 branch through inhibiting the degradation of bZIP17/28, resulting in the over‐accumulation of bZIP17/28. However, the molecular mechanisms about how the stability of unprocessed bZIP17/28 is regulated and how NSvc2 and NSvc4 interfere with the turnover of bZIP17/28 remain to be explored.

It has been speculated that the excessive viral proteins may not fold properly and accumulate in the ER, or some viral membrane‐associated proteins may change the structure of the ER membrane, which in turn affects ER homeostasis and activates the UPR (Verchot, 2016). In this study, however, RSV only activated the bZIP17/28 branch rather than the IRE1‐bZIP60 branch, while other viruses, such as TuMV, PVX and PlAMV, induce the splicing of *bZIP60* mRNA (Gaguancela et al., 2016; Gayral et al., 2020; Zhang et al., 2015), which suggests that the mechanisms of how plant viruses elicit the UPR may vary between different viruses and may not simply be attributed to the overaccumulation of unfolded proteins in the ER. Recently, Gayral et al. reported that *bZIP60* and *bZIP28* restrict the infection of TuMV, while *bZIP17* has no effect on viral infection; *bZIP60* and *bZIP28* redundantly support PlAMV infection, while *bZIP60* and *bZIP17* restrict PlAMV infection synergistically (Gayral et al., 2020). Therefore, we suspect that the two branches of the UPR signalling pathway may play different roles in virus infections, and there might be crosstalk between the two branches. Silencing NbbZIP17/28 delayed RSV infection and decreased the accumulation levels of viral RNA and protein (Figure 5a–d), which indicates that RSV can elicit the UPR through the bZIP17/28 branch specifically to promote its infection.

**FIGURE 6:**
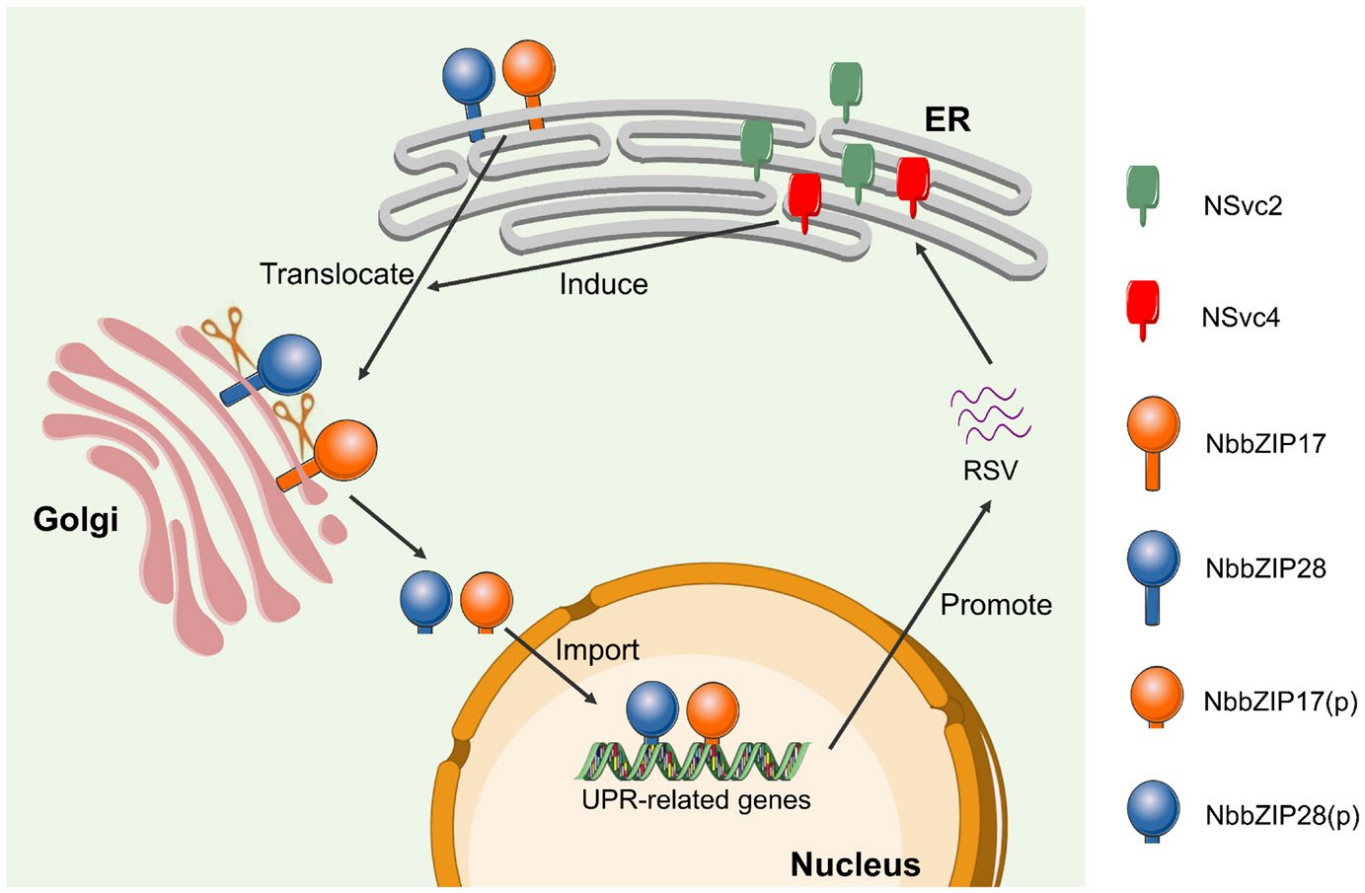
A working model for RSV eliciting the unfolded protein response (UPR) through the bZIP17/28 branch to promote viral infection. First, RSV‐encoded membrane‐associated proteins NSvc2 and NSvc4 localize at the endoplasmic reticulum (ER) membrane. Next, NSvc2 and NSvc4 induce the translocation of bZIP17 and bZIP28 from the ER membrane to the Golgi apparatus, where bZIP17/28 are proteolytically processed to remove the transmembrane domains (TMDs). Then, the processed bZIP17/28(p) are imported into the nucleus to up‐regulate the expression of UPR‐related genes, which facilitates viral infection

Here, we explored how RSV elicits the UPR and the function of the bZIP17/28 branch of the UPR signalling pathway upon RSV infection (Figure 6): RSV‐encoded membrane‐associated proteins NSvc2 and NSvc4 localize at the membrane of ER and induce the translocation of NbbZIP17/28 from the ER to the Golgi body in which NbbZIP17/28 are processed to generate the TMD‐removed NbbZIP17/28(p). Then, NbbZIP17/28(p) are introduced into the nucleus to regulate the expression of UPR‐related genes and promote virus infection. At present, three transcription factors are reported to be involved in the UPR signalling pathway in rice. Among them, *OsbZIP50* is an ortholog of *AtbZIP60* and is regulated by IRE1‐mediated splicing of its mRNA, while *OsbZIP39* and *OsbZIP60* are rice orthologs of *AtbZIP17* and *AtbZIP28*, respectively (Hayashi et al., 2012; Lu et al., 2012; Takahashi et al., 2012). However, whether OsbZIP39 or OsbZIP60 is selectively activated on RSV infection is still unclear and needs further investigation. Also, the exact mechanism of how NSvc2 and NSvc4 induce bZIP17/28 processing and whether the IRE1‐*bZIP60* branch is also involved in RSV infection remain to be determined in the future.

## 4 EXPERIMENTAL PROCEDURES

### 4.1 Plant materials, agroinfiltration, and virus inoculation

All plants used in this study were grown in a growth chamber set at 25°C, 60% relative humidity, and a 16 h light and 8 h dark photoperiod. Five‐leaf *N. benthamiana* plants were used for *Agrobacterium* infiltration, as described before (Fu et al., 2018). In brief, the expression vectors were transformed into *Agrobacterium tumefaciens* EHA105 by the electroporation method. Suspensions of *Agrobacterium* were adjusted to an OD_600_ of 0.6, while for Myc‐eGFP‐NbbZIP17/28A/28B the suspensions were diluted to OD_600_ of 0.04, 0.2, and 0.08, respectively. RSV mechanical inoculation was described previously (Fu et al., 2018; Kong et al., 2014).

### 4.2 Plasmid construction and RT‐PCR

The cDNA of RSV‐infected rice plants was generated by reverse transcription of total RNA, using ReverTra Ace qPCR RT Master Mix with gDNA Remover (TOYOBO) according to the manufacturer's instructions. Genes were cloned by PCR amplification with specific primers and fused into the corresponding vectors. The in planta expression vector pCP‐2×35S was digested by *Bam*HI and *Xho*I (Thermofisher), while NSvc2 and NSvc4 were fused into the vector using ClonExpress II One Step Cloning Kit (Vazyme) according to the manufacturer's instructions. *NbbZIP17*/*28* were cloned and fused with *eGFP* into the *Bam*HI‐ and *Xba*I‐digested pCP‐2×35S‐NMyc vector. The *NbbZIP60* CDS was cloned into *Bam*HI‐ and *Sal*I‐digested pCAMBIA1300‐2×35S‐GFP vector. For TRV‐based VIGS, a fragment combining 835–1049 bp of *NbbZIP17* ORF and 1717–1918 bp of *NbbZIP28A* ORF was constructed into the *Xba*I‐ and *Kpn*I‐digested TRV RNA2 vector. The sequences of primers are provided in Table S1.

### 4.3 Sequence analyses

The amino acid sequences of AtbZIP17 and AtbZIP28 were sent to BLASTp searching in the *N. benthamiana* draft genome sequence database (https://solgenomics.net/organism/Nicotiana_benthamiana/genome/) (Bombarely et al., 2012). Domain analysis was performed using InterProScan (http://www.ebi.ac.uk/InterProScan/) (Blum et al., 2021). Multiple full‐length protein sequence alignment (ClustalW method) and a maximum‐likelihood phylogenetic tree with 1000 bootstrap replicates were constructed using MEGA X software (https://www.megasoftware.net/) (Kumar et al., 2018). The secondary structure of mRNA was predicted using the mfold web server (Zuker, 2003).

### 4.4 RNA isolation and real‐time quantitative PCR

Total RNA was extracted from *N. benthamiana* leaf tissues using TRIzol reagent (Invitrogen) (Hu et al., 2020). Primer pairs specific to target genes were designed by Oligo 7 (https://www.oligo.net/). The cDNAs were generated using ReverTra Ace qPCR RT Master Mix with gDNA Remover, and qPCR was performed using LightCycler 480 (Roche) and ChamQ Universal SYBR qPCR Master Mix (Vazyme) as described previously (Fu et al., 2018).

### 4.5 The *bZIP60* splicing assay

The fragment of *NbbZIP60* cDNA was amplified through RT‐PCR using a primer pair flanking the 23‐bp intron (Table S1). PCR products were purified, and then 200 ng of the products was digested with *Alw*21I (Thermofisher) for 30 min at 37°C. Next, the digested and undigested products were separated by electrophoresis on 2% agarose gel, 0.5 × TBE running buffer.

### 4.6 Western blotting and antibodies

The extraction of plant total protein and western blotting was carried out as described previously (Xiong et al., 2009). In brief, the leaves were ground with liquid nitrogen, and 200 μl of the extraction buffer (9 M urea, 4.5% sodium dodecyl sulphate, 1.5% β‐mercaptoethanol, 50 mM Tris‐HCl pH 6.8) was added to 0.1 g samples. Next, the samples were centrifuged at 12,000 × *g* for 10 min and the resulting supernatants were incubated at 80°C for 5 min, then subjected for western blot analysis. The membranes were probed with specific primary antibodies against Myc tag (GenScript) or actin (ABclonal); the monoclonal antibody against RSV nucleocapsid protein (NP) was generated in our laboratory previously.

### 4.7 Chemical treatments

Tunicamycin (TM) was obtained from Sigma and dissolved in DMSO to a 5 mg/ml stock solution. Before using, the TM stock solution was diluted in double‐deionized water to the desired concentrations and an equal volume of DMSO diluted in double‐deionized water was used as control.

### 4.8 Subcellular localization assays and confocal microscopy

The eGFP fluorophore was excited at 488 nm and emission was detected at 490–540 nm. The RFP fluorophore was excited at 561 nm and emission was detected at 560–620 nm. The images were taken by a laser scanning confocal microscope FV3000 (Olympus) and processed with FV31S‐SW software (Olympus).

## CONFLICT OF INTEREST

The authors declare that they have no competing interest.

## Supporting information


**FIGURE S1.** The amino acid sequence alignment of bZIP17 (a) and bZIP28 (b) in *Arabidopsis thaliana* and *Nicotiana benthamiana*

**FIGURE S2.** Confocal images of *Nicotiana benthamiana* leaves expressing Myc‐eGFP‐NbbZIP17/28 at relatively high expression levels. The Myc‐eGFP‐NbbZIP17/28A/28B were expressed in *N. benthamiana* leaves through agroinfiltration (OD_600_ = 0.6). The images were taken at 48 h after agroinfiltration. RFP‐H2B was expressed as a nucleus marker

**FIGURE S3.** Reverse transcription quantitative PCR (RT‐qPCR) analysis of the unfolded protein response (UPR)‐related genes when expressing NS2, NS3, NP, SP. NS2, NS3, NP, SP or empty control in *Nicotiana benthamiana* leaves through agroinfiltration. The total RNA of the leaves samples was extracted for RT‐qPCR analysis at 60 h postinfiltration. *NbActin* served as an internal reference in relative quantification. The values represent the means of the expression levels ± *SD* relative to the empty vector control (*n* = 3 biological replicates). The values were analysed by analysis of variance followed by Dunnett’s test, and asterisks denote significant differences between viral proteins‐ and empty vector‐expressing leaves (two‐sided, *p < 0.05, **p < 0.01, ***p < 0.001)

**FIGURE S4.** Off‐target analysis of silencing *NbbZIP17*/*28* in *Nicotiana benthamiana*. The sequence inserted into the pTRV2 vector was analysed using the SGN VIGS Tool (https://vigs.solgenomics.net/) and the predicted targeted genes are shown

**TABLE S1.** Primers used in the study

## Data Availability

The data that support the findings of this study are available from the corresponding author upon reasonable request.
