# Supplementary figures and images for: Rice stripe virus activates the bZIP17/28 branch of the unfolded protein response signalling pathway to promote viral infection
Source: Mol Plant Pathol. 2021 Dec 11;23(3):447–58. doi: 10.1111/mpp.13171 (PMC8828695; doi:10.1111/mpp.13171)

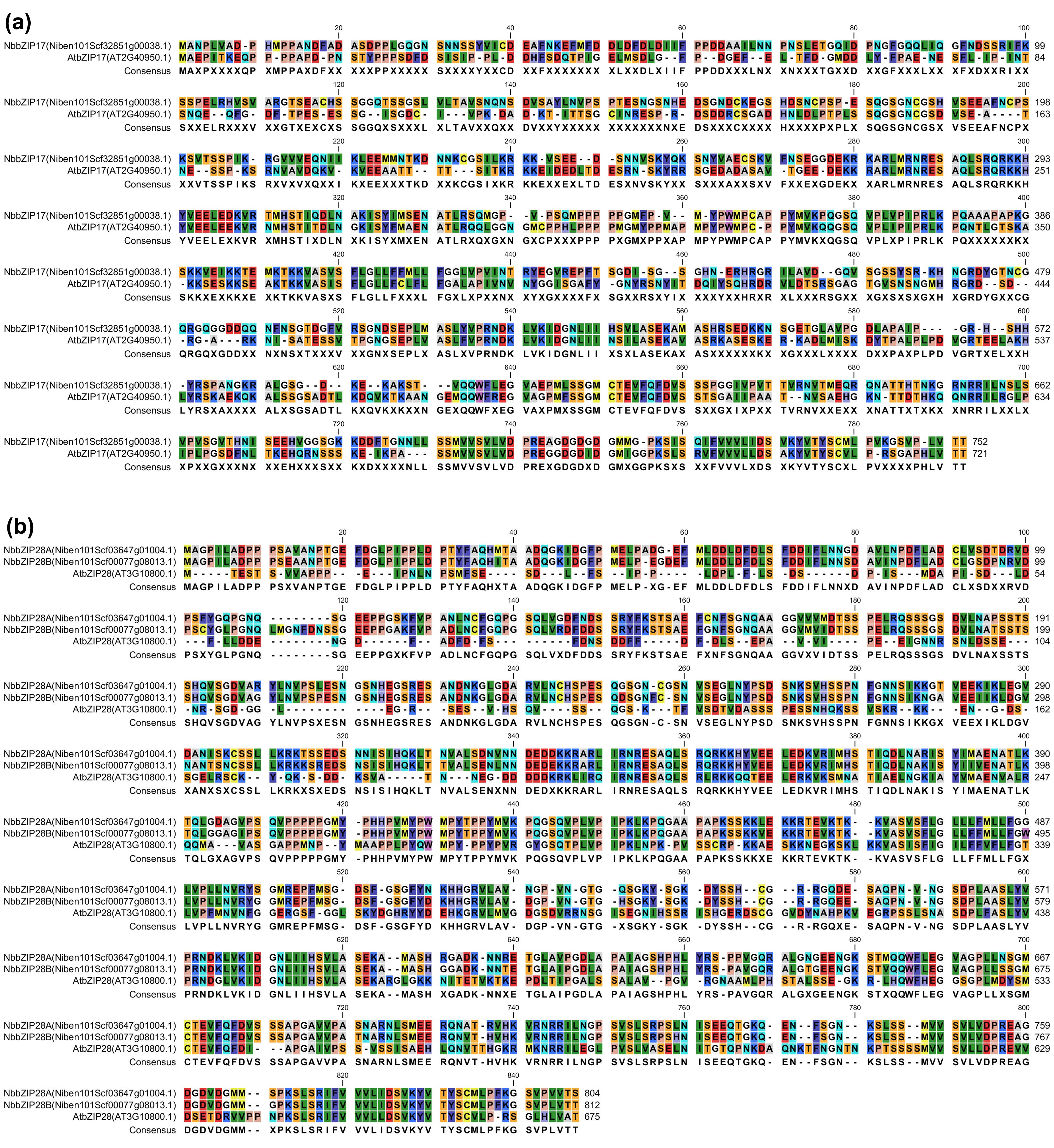

Supplement: Supplementary file 1 — FIGURE S1. The amino acid sequence alignment of bZIP17 (a) and bZIP28 (b) in Arabidopsis thaliana and Nicotiana benthamiana [file MPP-23-447-s003.tif]

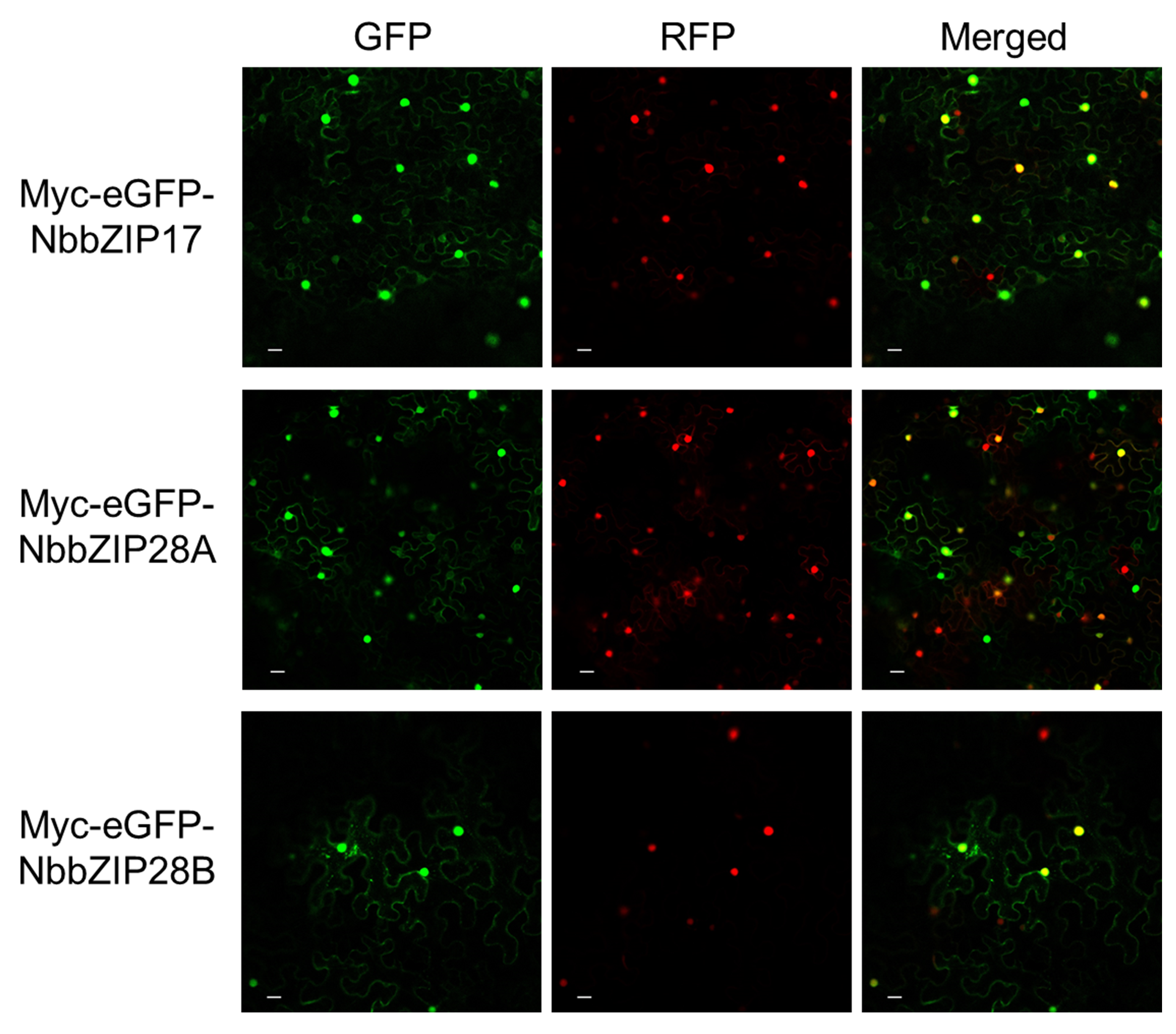

Supplement: Supplementary file 2 — FIGURE S2. Confocal images of Nicotiana benthamiana leaves expressing Myc‐eGFP‐NbbZIP17/28 at relatively high expression levels. The Myc‐eGFP‐NbbZIP17/28A/28B were expressed in N. benthamiana leaves through agroinfiltration (OD600 = 0.6). The images were taken at 48 h after agroinfiltration. RFP‐H2B was expressed as a nucleus marker [file MPP-23-447-s005.tif]

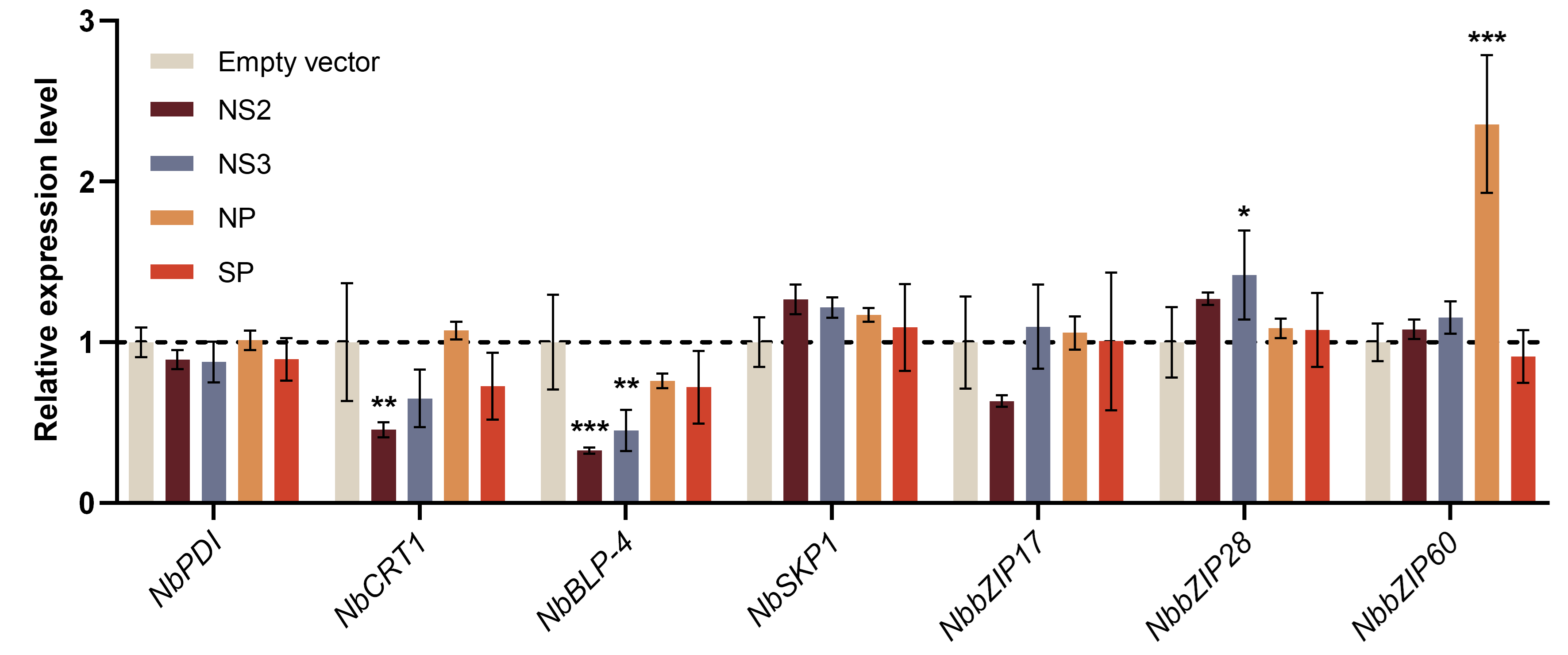

Supplement: Supplementary file 3 — FIGURE S3. Reverse transcription quantitative PCR (RT‐qPCR) analysis of the unfolded protein response (UPR)‐related genes when expressing NS2, NS3, NP, SP. NS2, NS3, NP, SP or empty control in Nicotiana benthamiana leaves through agroinfiltration. The total RNA of the leaves samples was extracted for RT‐qPCR analysis at 60 h postinfiltration. NbActin served as an internal reference in relative quantification. The values represent the means of the expression levels ± SD relative to the empty vector control (n = 3 biological replicates). The values were analysed by analysis of variance followed by Dunnett’s test, and asterisks denote significant differences between viral proteins‐ and empty vector‐expressing leaves (two‐sided, *p < 0.05, **p < 0.01, ***p < 0.001) [file MPP-23-447-s002.tif]

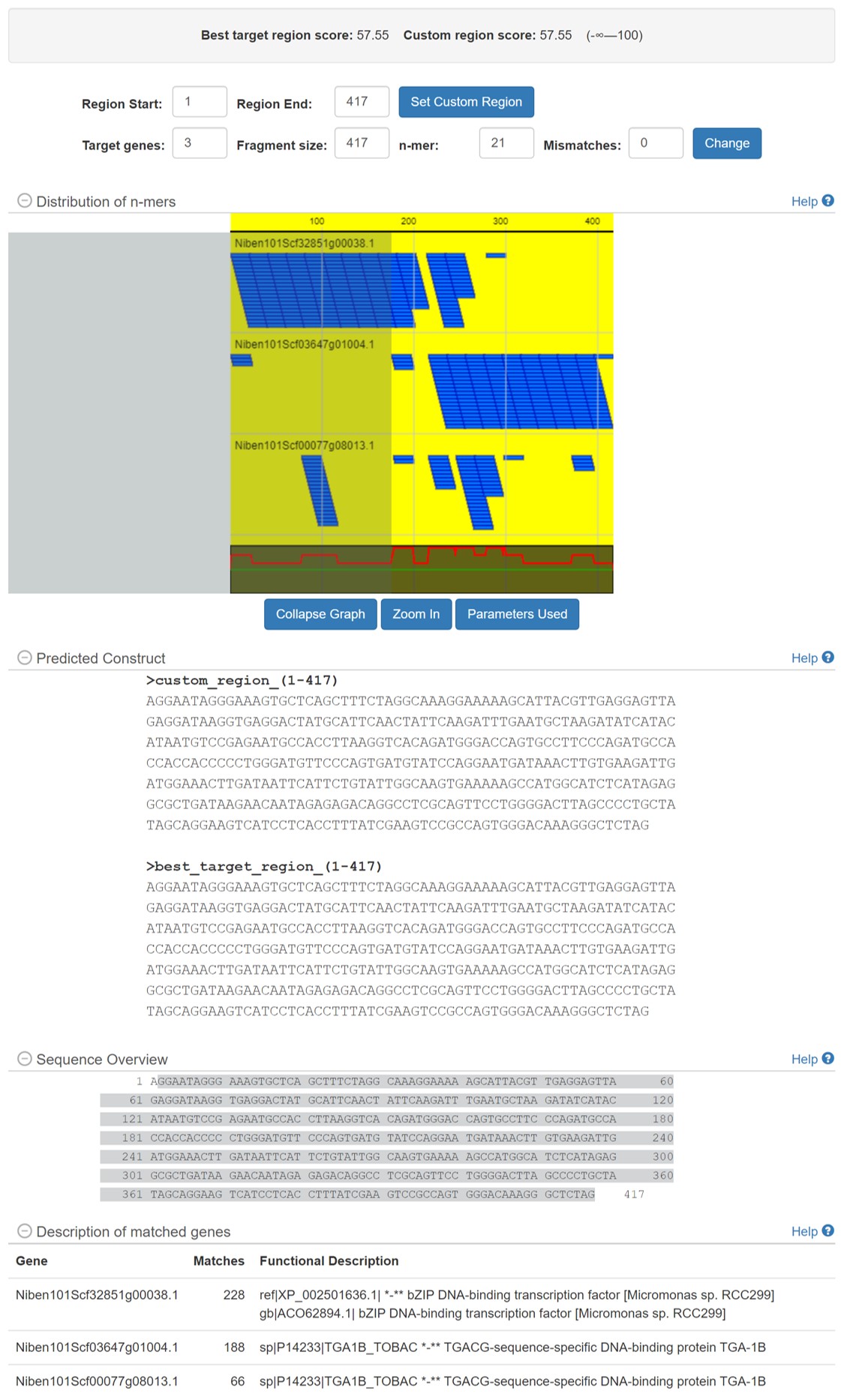

Supplement: Supplementary file 4 — FIGURE S4. Off‐target analysis of silencing NbbZIP17/28 in Nicotiana benthamiana. The sequence inserted into the pTRV2 vector was analysed using the SGN VIGS Tool (https://vigs.solgenomics.net/) and the predicted targeted genes are shown [file MPP-23-447-s004.jpg]
